# Drug Repurposing as an Antitumor Agent: Disulfiram-Mediated Carbonic Anhydrase 12 and Anion Exchanger 2 Modulation to Inhibit Cancer Cell Migration

**DOI:** 10.3390/molecules24183409

**Published:** 2019-09-19

**Authors:** Soyoung Hwang, Dong Min Shin, Jeong Hee Hong

**Affiliations:** 1 Department of Physiology, Lee Gil Ya Cancer and Diabetes Institute, College of Medicine, Gachon University, 155 Getbeolro, Yeonsu-gu, Incheon 21999, Korea; snrntlwy1004@gmail.com; 2 Department of Oral Biology, BK21 PLUS Project, Yonsei University College of Dentistry, Seoul 03722, Korea

**Keywords:** disulfiram (DSF), drug repurposing, antitumor agent, cancer cell migration

## Abstract

Disulfiram has been used in the treatment of alcoholism and exhibits an anti-tumor effect. However, the intracellular mechanism of anti-tumor activity of Disulfiram remains unclear. In this study, we focused on the modulatory role of Disulfiram via oncogenic factor carbonic anhydrase CA12 and its associated transporter anion exchanger AE2 in lung cancer cell line A549. The surface expression of CA12 and AE2 were decreased by Disulfiram treatment with a time-dependent manner. Disulfiram treatment did not alter the expression of Na^+^-bicarbonate cotransporters, nor did it affect autophagy regulation. The chloride bicarbonate exchanger activity of A549 cells was reduced by Disulfiram treatment in a time-dependent manner without change in the resting pH level. The expression and activity of AE2 and the expression of CA12 were also reduced by Disulfiram treatment in the breast cancer cell line. An invasion assay and cell migration assay revealed that Disulfiram attenuated the invasion and migration of A549 cells. In conclusion, the attenuation of AE2 and its supportive enzyme CA12, and the inhibitory effect on cell migration by Disulfiram treatment in cancer cells provided the molecular evidence supporting the potential of Disulfiram as an anticancer agent.

## 1. Introduction

Disulfiram (DSF) has been used in the treatment of alcoholism as a proteasome inhibitor. DSF inhibits acetaldehyde dehydrogenase involved in the oxidation of ethanol metabolite acetaldehyde [1]. In addition, DSF binds to metal copper ion and binding complex DSF -copper has been reported to exhibit anti-tumor effect. The binding complex generates reactive oxygen species, which trigger cancer cell apoptosis [2]. DSF also chelated zinc ion, and subsequently inhibited the proteolytic activity of matrix metalloproteinases (MMP), MMP-2 and MMP-9 [3], and is potentially involved in the inhibition of cancer cell invasion and angiogenesis [4].

Plasma membrane-associated carbonic anhydrases 12 (CA12) can be considered as a cancer cell surface marker [5,6,7] and exhibits a significant relationship in various cancers, such as breast cancer [8,9]. In addition to being a novel prognostic indicator, modulation of CA12 exhibits a novel therapeutic strategy for cancer. Previously, we reported that the involvement of CA12 enhanced several bicarbonate transporters activities [10] and mutated CA12 modulated the expression of water channel aquaporin 5 [11]. The modulatory role of CA12, ion transporters, and its coordinated arrangement is involved in the maintenance of acidic tumor microenvironment [12].

In this study, we focused on the anti-invasion effect and modulatory role of DSF via CA12 and its associated transporter anion exchanger 2 (AE2). The upregulation of AE2 as a chloride/bicarbonate exchanger (CBE) is associated with various cancer cell types, including hepatocarcinoma [13,14], gastric cancer [15,16], and ovarian cancer [17]. Cellular migration and invasion are modulated by the involvement of various ion transporters [18]. We hypothesized that the acidic tumor microenvironment and surface-associated CA12 are also associated with the recruitment of transporters. In addition, DSF -induced modulation of cancer cell invasion might be mediated by alteration in expression or activity of CA12 and AE2. Thus, we examined the role of DSF on the modulation of CA12 and AE2 and, for the first, demonstrated the inhibitory effect of DSF on cancer cell invasion and AE2 activity.

## 2. Results

### 2.1. Surface Expression of CA12 Protein was Decreased by DSF Treatment 

**Figure 1:**
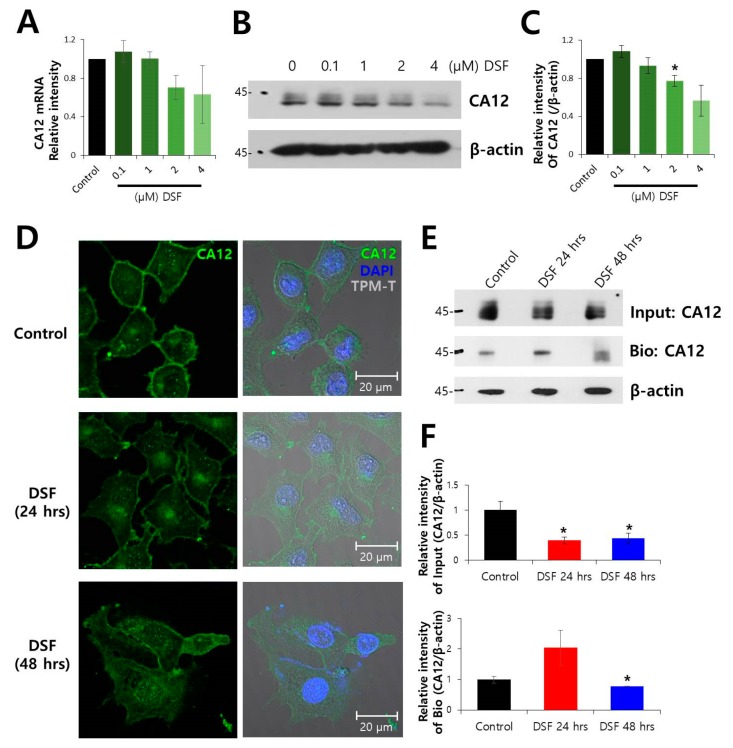
Surface protein expression of CA12 protein was decreased by DSF treatment. (**A**) Analysis of CA12 mRNA expression at rising concentrations from 0.1–4 μM DSF for 48 h (n = 5). (**B**) Protein expression of CA12 at indicated DSF concentration. (**C**) Analysis of relative CA12 intensity (n = 3, * *p* < 0.05). (**D**) Immunostained images of CA12 (green), nucleus (DAPI, blue), and cell morphology (TPM-T, gray) following 2 μM DSF treatment. The scale bars represent 20 μm. (**E**) Time-dependent changes in surface expression of CA12 in the presence of 2 μM DSF in A549 cells. The β-actin and input CA12 blots were used as loading controls. (**F**) Analysis of relative intensity of CA12 for input and biotinylated (Bio) blot. Bars represent mean ± SEM (n = 4, * *p* < 0.05).

CA12 can be considered as a cancer cell surface marker [5,6,7]. To determine the dose of DSF on CA12 expression, the expression of mRNA and protein was examined at rising concentrations from 0.1–4 μM DSF (Figure 1A–C). The 2 μM DSF was selected for further investigations. To evaluate the effect of DSF on CA12 expression in a time-dependent manner, DSF-treated cells were immunostained with native CA12. The CA12 is localized in the plasma membrane and the expression of CA12 was decreased by DSF treatment in a time-dependent manner (Figure 1D). The biotinylation assay also revealed reduced the surface expression of CA12 by DSF (Figure 1E,F). The viability of cells in the presence of DSF was confirmed with MTT assay and FACS analysis (Supplementary Figure S1A–C). The treatment with DSF under this condition did not affect the cell viability. 

### 2.2. Surface Expression of AE2 Protein was Decreased by DSF Treatment 

**Figure 2:**
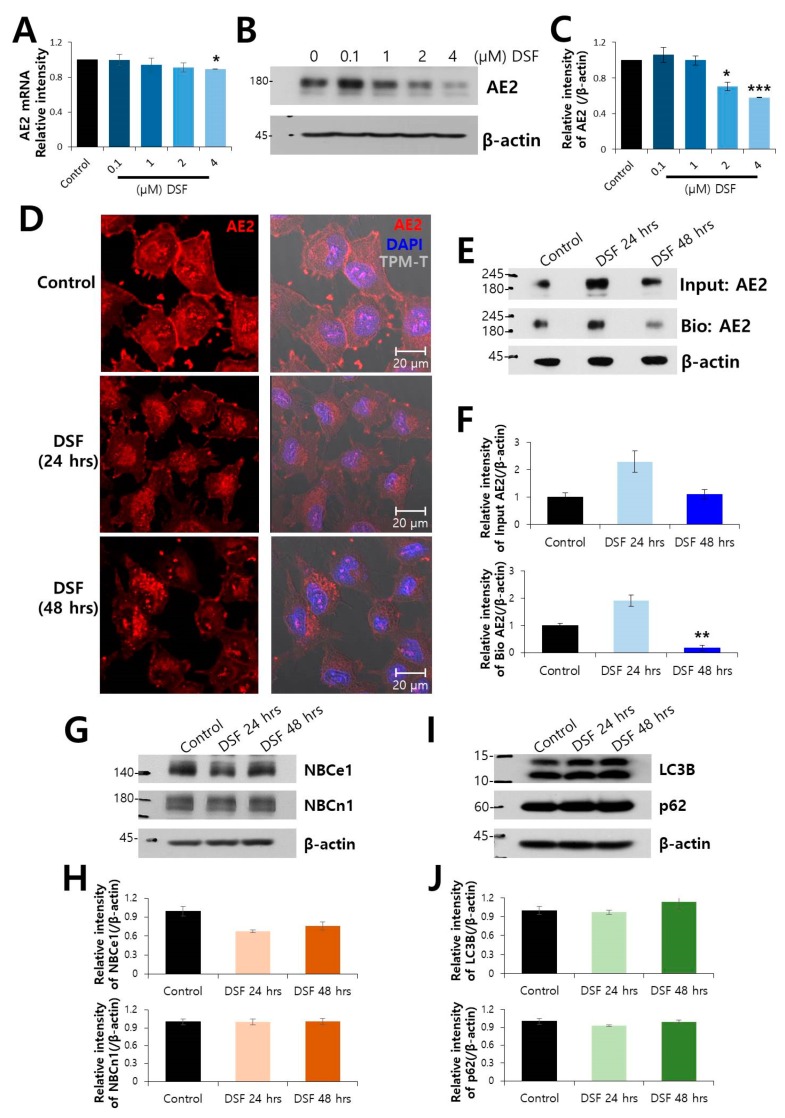
Surface protein expression of AE2 protein was decreased by DSF treatment. (**A**) Analysis of AE2 mRNA expression at rising concentrations from 0.1–4 μM DSF for 48 h (n = 5). (**B**) Protein expression of AE2 at indicated DSF concentration. (**C**) Analysis of relative AE2 intensity (n = 3, * *p* < 0.05, *** *p* < 0.001). (**D**) Immunostained images of AE2 (red), nucleus (DAPI, blue) and merge of cell morphology (TPM-T, gray) with and without DSF treatment. The scale bars represent 20 μm. (**E**) The surface expression of AE2 after treatment with 2 μM DSF. The β-actin and input AE2 blots were used as loading controls. (**F**) Analysis of relative intensity of AE2 for input and biotinylated (Bio) blot. Bars represent mean ± SEM (n = 3, ** *p* < 0.01). (**G**) Protein expressions of NBCe1 and NBCn1. (**H**) Analysis of relative intensity of NBCe1 and NBCn1 blots. Bars represent mean ± SEM. (**I**) Protein expressions of LC3B and p62. The β-actin blot was used as a loading control. (**J**) Analysis of relative intensity of LC3B and p62 blots. Bars represent mean ± SEM.

We have previously shown the interaction between CA12 and chloride/bicarbonate exchanger AE2 [10,19]. Here, we verified the effect of DSF on the levels of AE2. To confirm the dose of DSF on AE2 expression, expression of mRNA and protein was examined at 0.1–4 μM DSF (Figure 2A–C). More than 2 μM DSF revealed statistical significance at the reduced expression of endogenous AE2 compared to control. The immunostaining of endogenous AE2 revealed its localization on plasma membrane and decreased membrane localization of AE2 following DSF treatment (Figure 2D). The surface expression of endogenous AE2 was also decreased by treatment with DSF (Figure 2E,F). To confirm the effect of DSF on the level of AE2, we used overexpressed AE2 in HEK293T cells. The surface expression of overexpressed AE2 also was decreased by the treatment with DSF (Supplementary Figure S2). In addition, Na^+^-dependent bicarbonate transporters are also involved in the cell migration [20,21]. However, DSF treatment did not alter the expression of electrogenic Na^+^-bicarbonate cotransporter (NBC)e1 and electroneutral NBCn1 (Figure 2G,H). It has been previously reported that the expression of AE2 was associated with autophagy regulation [22]. We examined the expression of autophagy marker proteins. The expression of LC3B and p62 did not change in DSF-treated cells (Figure 2I,J).

### 2.3. DSF Decreased CBE Activity in A549 Cells 

**Figure 3:**
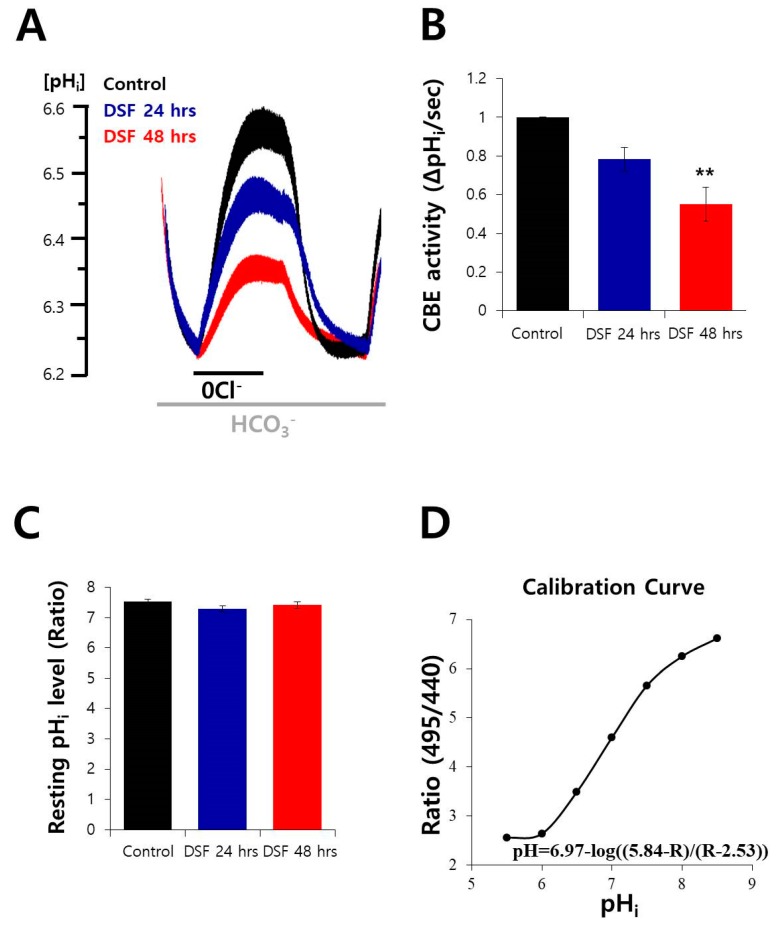
DSF decreased CBE activity in A549 cells. (**A**) CBE activity of A549 cells with and without treatment with 2 μM DSF (blue trace at 24 h, red trace at 48 h, and control, black trace). Average traces are represented. (**B**) Bars indicate the means ± SEM (n = 4, ** *p* < 0.01). (**C**) The bars show resting intracellular pH (pH_i_) level of A549 cells represented as the means ± SEM. (**D**) The calibration curve of A549 cells (pH = 6.97-log(5.84-R/R-2.53)).

We verified the effect of DSF on CBE activity. The CBE activity of A549 cells was reduced after DSF treatment in a time-dependent manner (Figure 3A,B). The DSF treatment did not affect the resting pH level (Figure 3C). As shown in Figure 3D, the changes in BCECF ratios was converted to intracellular pH value followed by the calibration curve of A549 cells (pH = 6.97-log(5.84-R/R-2.53)).

### 2.4. DSF Decreased the Expression of AE2 and CA12 and CBE Activity in Breast Cancer Cell Lines 

**Figure 4:**
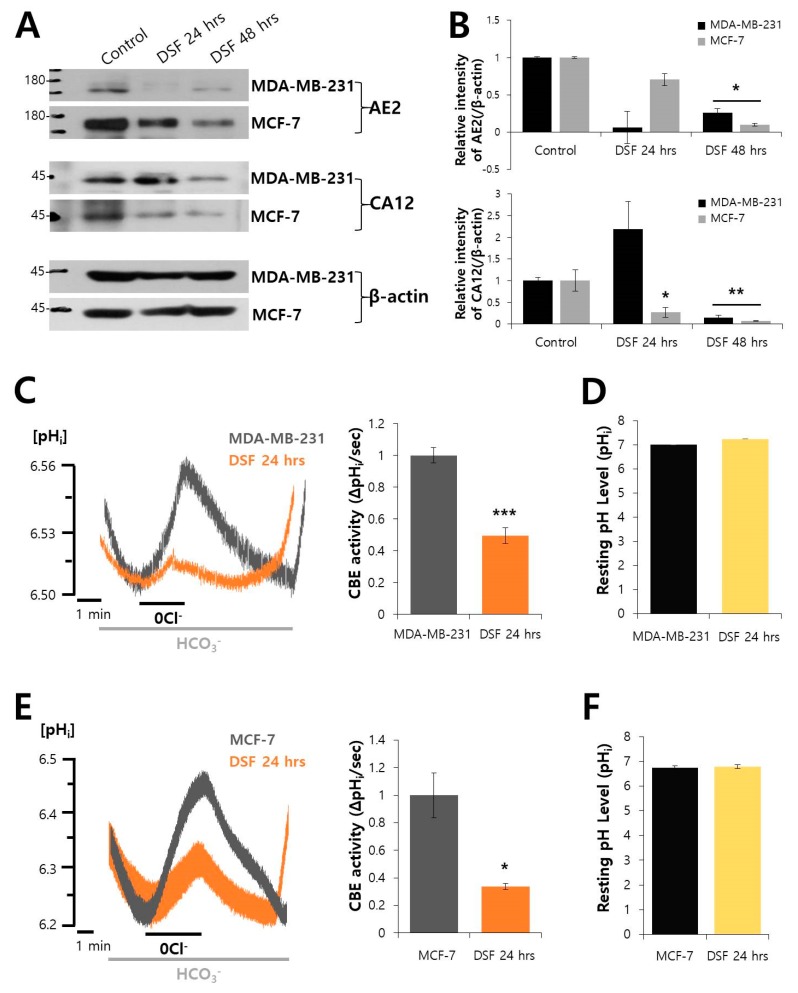
DSF also decreased the expressions of AE2 and CA12 and CBE activity in breast cancer cell lines. (**A**) The protein expressions of AE2 and CA12 with and without treatment of 2 μM DSF in MDA-MB-231 and MCF-7 cells. The β-actin was used as loading control. (**B**) Analysis of relative intensity of AE2 and CA12 blots in MDA-MB-231 and MCF-7 cells. Bars represent mean ± SEM (n = 3, * *p* < 0.05, ** *p* < 0.01). (**C**) CBE activity of MDA-MB-231 cells treated with 2 μM DSF (yellow trace) at 24 h and the cells not treated with DSF (Control, gray trace). Average traces are represented. The bar graph indicates CBE activity of MDA-MB-231 cells with and without DSF and the means ± SEM (n = 4, *** *p* < 0.001). (**D**) The bars show resting pH_i_ level of MDA-MB-231 cells which indicate the means ± SEM. (**E**) CBE activity of MCF-7 cells treated with 2 μM DSF (yellow trace) at 24 h and the cells not treated with DSF (Control, gray trace). Average traces are represented. The bar graph indicates CBE activity of MCF-7 cells with and without DSF and the means ± SEM (n = 4, * *p* < 0.05). (**F**) The bars show resting pH_i_ level of MCF-7 cells which indicate the means ± SEM.

To evaluate the inhibitory role of DSF on other types of cancer, breast cancer cell lines, MDA-MB-231 and MCF-7, were treated DSF in a time-dependent manner. The expressions of AE2 and CA12 were also reduced by DSF treatment (Figure 4A,B). CBE activities of MDA-MB-231 and MCF-7 cells were also inhibited by the treatment of DSF (Figure 4C,E). DSF treatment did not affect the resting pH level in MDA-MB-231 and MCF-7 cells (Figure 4D,F). The changes in BCECF ratio were converted to intracellular pH value followed by the calibration curve of each cell line (Supplementary Figure S3).

### 2.5. DSF Reduced Cancer Cell Invasion and Migration in Acidic Microenvironment 

**Figure 5:**
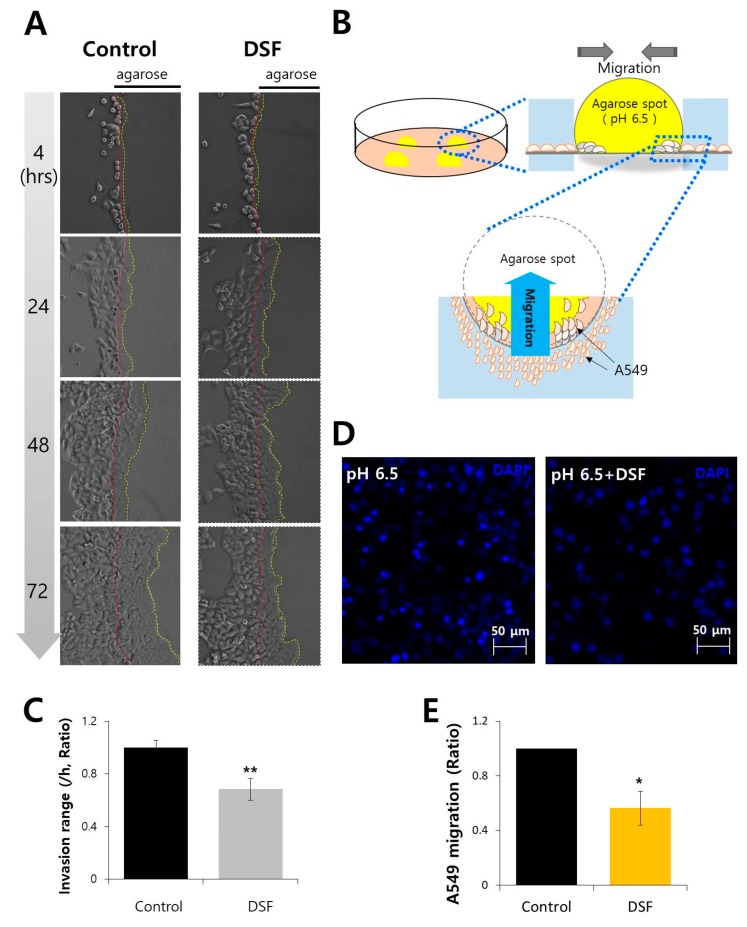
DSF reduced cancer cell invasion and migration in acidic microenvironment. (**A**) Time dependent representative images of A549 cells, invading (4, 24, 48, and 72 h) towards agarose spots containing PBS (pH 6.5) with and without DSF treatment. The red dotted lines indicate the direction of invasion across the boundary of the agarose spot shown as a dashed curve. The yellow dotted lines indicate the lineage of cells that migrated into the agarose spots. (**B**) The schematic representation of agarose spot assay. (**C**) Analysis of invasion range per hour. Bars indicate the means ± SEM (n = 4, ** *p* < 0.01). (**D**) A549 cell migration was measured by transwell-membrane migration assay incubated with indicated conditions (bottom plates; pH 6.5, upper chamber; pretreated with 2 μΜ DSF at 48 h). Immunofluorescence staining of nucleus (DAPI, blue) was analyzed for the evaluation of cell migration. The scale bars represent 50 μm. (**E**) Analysis of total intensity of DAPI for A549 migration. Bars represent mean ± SEM (n = 3, * *p* < 0.05).

The localization of CA12 in plasma membrane provides favorable circumstances for cancer cell migration. The ability of invasion and migration was mediated by transporters. Thus, we hypothesized that the attenuation of AE2 activity by DSF affects the motility of cells. We examined the effect of DSF on the invasion and migration of A549 cells. For invasion assay, cells were seeded on the agarose spot-containing culture dishes. A549 cells invaded towards agarose spots (Figure 5A). The schematic procedure was represented in Figure 5B. The invasion range decreased in DSF -treated cells (Figure 5C). The effect of DSF on cell migration was analyzed using transwell-membrane migration assay (Figure 5D). The cells migrated from upper chamber to bottom plates and migrated cells were placed in the porous membrane of the upper chamber. Cell migration was analyzed to determine by evaluating the number of nucleus with 4′,6-diamidino-2-phenylindole (DAPI) staining that represented migrated cells. A549 cell migration was also reduced by DSF treatment (Figure 5E). These results showed that DSF attenuated the invasion and migration of A549 cells.

## 3. Materials and Methods

### 3.1. Immunofluorescence and Confocal Imaging

The A549 cells was transferred onto cover glasses and fixed with chilled methanol (−20°C, 10 min for AE2) and 4% paraformaldehyde (room temperature (RT), 10 min for CA12). For the paraformaldehyde fixation, cells were treated 0.5% Triton-X 100 for 10 min. Fixed cells were treated with 5% goat serum (in DPBS) for 1 hr at RT to block non-specific reaction. The cells were incubated with primary antibodies (AE2 and CA12, 1:100 dilution factor in 5% goat serum) at 4 °C for overnight, followed by washing with incubation buffer (5% BSA in DPBS). To detect the primary antibody, cells were treated with secondary antibodies (1:200 dilution factor), goat immunoglobulin G (IgG)-tagged with rhodamine (1:50 dilution factor in incubation buffer, Jackson ImmunoResearch, anti-mouse: 115-025-072, anti-rabbit: 111-025-144) or fluorescein isothiocyanate (FITC, 1:50 dilution factor in incubation buffer, anti-mouse: 115-095-071, anti-rabbit: 111-095-003) for 1 hr at room temperature (RT). Following incubation, cells were washed thrice with PBS and the cover glasses were mounted on glass slides using 20 μL Fluoromount-G^TM^ with 4′,6-diamidino-2-phenylindole (DAPI, 17984-24, Electron Microscopy Sciences, Hatfield, PA, USA) and incubated overnight at 4 °C. The immunostaining images were detected using LSM 700 Zeiss confocal microscope (Carl Zeiss, Oberkochen, Germany).

### 3.2. Agarose Spot Assay for Cell Invasion

Directional cell invasion was examined by performing an agarose spot assay as described previously [23] with mild modifications. Briefly, 10 mg of agarose (UltraKem LE, Young Sciences, Bucheon, South Korea) was placed into 50 mL conical tube and diluted using 2 mL Physiological salt solution (called PSS, 140 mM NaCl, 10 mM HEPES, 10 mM Glucose, 5 mM KCl, 1 mM MgCl_2_, 1 mM CaCl_2_, 300mOsm, pH 7.4) to prepare a 0.5% agarose solution and spotted (four spots per plate) onto six-well plates (Thermo Fisher Scientific, Waltham, MA, USA) and allowed to cool for 8 min at 4 °C. Then, 4 × 10^5^ cells were plated and allowed to adhere for 4 h before replacement with a medium containing 0.1% fetal bovine serum (FBS; 1600-044, Invitrogen, Carlsbad, CA, USA) and 100 U/mL penicillin (15140122, Invitrogen, Carlsbad, CA, USA) in DMEM. After incubation for 4, 24, 48, and 72 h at 37 °C, images were collected using Meta Morph software (Molecular Devices, San Jose, CA, USA) with 20 x objective lens (Olympus, Tokyo, Japan). Invasive cells referred to the cells that appeared underneath the agarose spot—their distance of movement per hour was measured to evaluate the migration range.

### 3.3. Analysis of Cell Motility on Transwell System

First, 500 μL of A549 cell culture (5 × 10^4^ cells) was added to each bottom plate for 12~18 h. After incubation, top chambers were filled with 200 μL of A549 (5 × 10^4^ cells) containing 1% FBS and reagents for 6 h. Then, chilled methanol was added on the bottom plates for 1 min at -20°C. The chilled methanol was removed and PBS was added for washing. Then, DAPI solution, mixed with distilled water (DW), was loaded on the bottom plates. The plate was incubated for 30 min in dark. The media on the top and bottom plates was removed carefully. DW was added in the bottom plates at RT and measured at 340 nm using an LSM 700 confocal laser scanning microscope (Carl Zeiss, Oberkochen, Germany). A549 cell migration was determined by evaluating the number of nucleus with DAPI staining on the transwell-membrane. 

### 3.4. DNA Transfection

Plasmid DNA transfection was performed by Lipofectamine 2000 as per manufacturer’s protocol (Invitrogen, Carlsbad, CA, USA). Each plasmid DNA was diluted in 200 μL of Opti-MEM and 4 μL of Lipofectamine 2000, and was incubated for 5 min at RT with 200 μL of the same medium. The DNA samples and Lipofectamine 2000 were mixed and added to the cell culture dish after the incubation for 25 min. After further 4 h incubation, the medium was replaced with fresh DMEM containing FBS. The cells were cultured and used for experiment after 48 h of transfection.

### 3.5. Reverse Transcription-polymerase Chain Reaction (RT-PCR) 

Total RNA was extracted from A549 cells using the RiboEx™ (GeneAll, Seoul, South Korea), as per the manufacturer’s instructions. RNA concentrations were quantitated with the Spectrophotometer ND-1000 (Thermo Fisher Scientific, Waltham, MA, USA) and were amplified according to manufacturer’s protocol using the Hybrid-R™ (GeneAll, Seoul, South Korea) RT-PCR kit. The human primers used are listed; GAPDH (forward, 5′-CAT GGC ACC GTC AAG GCT GAG-3′ and reverse, 5′-CTT GGC CAG GGG TGC TAA GC-3′), CA12 (forward, 5′-CGT GCT CCT GCT GGT GAT CT-3′ and reverse, 5′-AGT CCA CTT GGA ACC GTT CAC T-3′), and AE2 (forward, 5′-GGG GAC AAG CCC AAG ATT CA-3′ and reverse, 5′-CTC GGT CGG TGA AGA TAC GG-3′). The PCR cycling protocol was as follows; denaturation at 95°C for 5 min, followed by 45 cycles of 95 °C for 1 min, an annealing step for 1 min, and an extension step at 72 °C for 1 min. Final extension was carried out at 72°C for 10 min. PCR products were electrophoresed on 1% agarose gels. Bands were visualized and acquired with a CCD camera, and scanned using the GelDoc^XR^ imaging system (BioRad, Hercules, CA, USA).

### 3.6. Surface Biotinylation and Western Blotting

To detect the surface expression of proteins, cells were incubated with 0.5 mg/mL EZ-LINK Sulfo-NHS-LC-biotin (21335, Thermo Fisher Scientific, Waltham, MA, USA) for 30 min on ice, followed by their treatment with 100 mM cold glycine solution for 10 min. Incubated cells were washed with phosphate-buffered saline. The cells were incubated with 1x lysis buffer (Cell signaling, Danvers, MA, USA) containing 20 mM Tris, 150 mM NaCl, 2 mM EDTA, 1% Triton X-100, and a protease inhibitor mixture for 30 min and subjected to sonication. The lysed cells were centrifuged at 11,000× *g* for 15 min at 4 °C and protein concentration was determined by Bradford assay (5000001, BioRad, Hercules, CA, USA). The supernatants were incubated overnight with 90 μL Avidin beads (20347, Thermo Fisher Scientific, Waltham, MA, USA) at 4 °C, followed by washing of the beads with lysis buffer. The collected beads were incubated with protein sample buffer at 37 °C for 15 min to recover the proteins. The recovered protein samples (30 μg) were subjected to separation using sodium dodecyl sulfate polyacrylamide gel electrophoresis (SDS-PAGE), and then, transferred onto polyvinylidene difluoride (PVDF, 1620177, BioRad, Hercules, CA, USA) membranes soaked in methanol. The membrane was blocked with 5% non-fat milk solution in TBS-T (Tris-buffered saline [TBS] and 0.5% Tween-20) for 1 hr. The membrane was then incubated with β-actin (A5441, Sigma, Saint-Louis, MO, USA), AE2 (ab42687, Abcam, Cambridge, UK), NBCe1 (ab187511, Abcam, Cambridge, UK), NBCn1 (ab82335, Abcam, Cambridge, UK), LC3B (NB100-2220, Novus Biologicals, Centennial, CO, USA), p62 (ab56416, Abcam, Cambridge, UK), and CA12 (15180-1-AP, Proteintech Group Inc, Rosemont, IL, USA) antibodies overnight at 4 °C and washed thrice with TBS-T. The membranes were incubated with horseradish peroxidase-conjugated anti-mouse and anti-rabbit secondary antibodies and the protein bands were visualized using the enhanced luminescent solution (BioRad, Hercules, CA, USA). 

### 3.7. Measurement of CBE Activity

Cells were attached onto coverslips one day before and loaded in the chamber with 4 μM BCECF-AM (2′,7′-bis-(carboxyethyl)-5-(and-6)-carboxyfluorescein, 0061, TEFlabs Inc, Austin, TX, USA) in the presence of same volume of 0.05% pluronic acid (P-3000MP, Invitrogen, Carlsbad, CA, USA) for 15 min at RT. After incubation of the fluorescence dye, the cells were perfused with regular solution, as previously described [24], for at least 5 min prior to intracellular pH (pH_i_) measurements. The pH_i_ was measured by BCECF fluorescence using dual excitation wavelengths of 495 and 440 nm and emission wavelength of 530 nm. The cells were incubated with CO_2_-saturated HCO_3_^−^ solution for the acidification of the cytosol, and then, perfused with Cl^−^-free HCO_3_^−^ solution. All incubated solutions were maintained at 37°C. Cl^−^-HCO_3_^−^ exchanger (CBE) activity of AE2 was calculated from the slope of increase in pH_i_ during the first 30-45 sec in Cl^−^-free HCO_3_^−^ solution and expressed as the percent fold change relative to that of CBE activity of control. Fluorescence images were obtained with Retiga 6000 CCD camera (Q-Imaging) recruited to an inverted microscope (Olympus, Tokyo, Japan) and analyzed with a Meta Fluor system (Molecular Devices, San Jose, CA, USA). Each image was analyzed after the removal of the background fluorescence. 

### 3.8. pH Calibration

Ratios of BCECF-AM (TEFLabs Inc, Austin, TX, USA) were converted to pH unit using calibration curves as previously described [25,26]. Briefly, pH calibration was performed by aspirating the calibration solution slowly to A549 cells attached on coverslips. Cells were incubated in the calibration solutions (pH 5.5, 6.0, 6.5, 7.0, 7.5, 8.0, and 8.5) for 5 min at RT. The equation of pH calibration curve was pH = 6.97 − log((R_max_ [5.84]-R)/(R-R_min_ [2.53])) (pKa of BCECF; 6.97, R; ratio value of BCECF, R_max_ [5.84]; maximum ratio, R_min_ [2.53]; minimum ratio) of A549 cells. The BCECF fluorescence ratio was converted to changes in pH_i_ (ΔpH_i_) value, followed by the calibration curve.

### 3.9. MTT Assay for Cell Viability

A549 cells were cultured in 96-well plates at a density of 5 × 10^3^ cells per well. The cells were treated with DSF (D2950000, Sigma, Saint-Louis, MO, USA). After the indicated incubation time, the medium was replaced with MTT (2 mg/mL, MT1036, BioPrince, Chuncheon, South Korea) solution followed by incubation for 2 h at 37 °C. The formazan crystals were dissolved by replacing with DMSO. The absorbance was determined using a Fluorescence micro-plate reader (VICTOR X3, PerkinElmer, Waltham, MA, USA) at 570 nm.

### 3.10. FACS Analysis

For analyzing apoptotic pathways in A549 cells treated with DSF, cells were seeded in six-well plates at a density of 3 × 10^5^ cells and incubated for the indicated time. Cell suspension with no DSF treatment was used as control. Apoptosis was assessed using an LSR-II flow cytometer (Invitrogen, Carlsbad, CA, USA). Cells were stained with annexin V using an apoptosis detection kit. After incubation, cells were detached from the culture dish and prepared according to the protocol supplied with the Annexin V-Pacific Blue™ kit (Invitrogen, Carlsbad, CA, USA). Briefly, 5 μL of Annexin V-Pacific Blue™ was applied to each sample and incubated for 15 min in the dark at RT. Then, cells were re-suspended in 400 μL of PBS containing 10% FBS, and analyzed at 410 nm excitation using a 455 nm filter to measure Pacific Blue.

### 3.11. Statistical Analyses

Data from the indicated number of experiments were expressed as mean ± standard error of the mean (SEM). Statistical significance was determined by the analysis of variance in each experiment (* *p* < 0.05, ** *p* < 0.01, *** *p* < 0.001).

## 4. Discussion

In this study, we reported, for the first time, that DSF modulated endogenous CA12 and endogenous AE2 and attenuated the migration and invasion of cancer cells by reduction of expression and activity of AE2. Broadly expressed AE2 is upregulated in several tumor types, such as hepatocellular carcinoma [13,14], gastric cancer [15,16], and ovarian cancer [17]. The involvement of ion transporters reveals enhanced cell motility [18]. In addition to motility, the acidic or hypoxic tumor microenvironments are needed for sophisticated pH regulation for cancer progression. Tumor microenvironment was regulated by the acid-base balancing mechanism. As diagnostic markers, CA9 and CA12 have been shown to be tumor hypoxia markers [27,28]. Thus, the involvement of bicarbonate transporters and associated enzymes, such as CA9 and CA12, provides favorable condition to facilitate cancer progression as well as motility [21,29].

Cellular migration is modulated by various transporters, including NBCs, Na^+^K^+^Cl^−^ cotransporter, and AE [20,21]. We previously reported that CA12 is associated with NBC and AE2 [10]. Thus, we assumed that DSF treatment could inhibit NBC and AE. Interestingly, DSF treatment did not inhibit Na^+^-bicarbonate cotransporters as shown in Figure 2G,H. It indicated that DSF prominently regulated both CA12 and AE2. We previously reported the supportive function of CA12 on the regulation of AE2 activity [10]. The current results have addressed that co-modulation of CA12 and AE2 by DSF may provide effective strategy to attenuate the migration of cancer cells. Specific inhibitors or si/shRNAs of AE2 and/or CA12 may mimic the attenuated effect of DSF on migration.

It was previously shown that decreased AE2 expression was related to autophagy dysregulation [22]. In this study, attenuation of AE2 by DSF was independent of autophagic regulation. Accumulating evidence suggests that AE2 is involved in tumorigenesis. Celay et al. addressed the role of AE2 and its target peptide reduced tumor growth in vivo [30]. Enhanced AE2 promotes ovarian cancer progression by activating mTOR signaling [17]; whereas, reduction of AE2 enhances cellular migration via the activation of MMP pathways in the esophageal squamous cell carcinoma [31]. Despite the controversial effect of AE2 on tumorigenesis, as a drug repurposing, the attenuation of AE2 and its supportive enzyme CA12, and the inhibitory effect on cell migration by DSF in lung and breast cancer cells, provides evidence supporting the therapeutic potential of DSF against cancer as well as alcoholism.

## Supplementary Materials

The following are available online, Supplementary Figure S1. Cell viability of DSF-treated A549 cells. Supplementary Figure S2. The effect of DSF on the localization of AE2 in AE2-overexpressed HEK293T cells. Supplementary Figure S3. Calibration curve of intracellular pH change (ratio) of MDA-MB-231 and MCF-7 cells.
