# Supplementary material for: Drug Repurposing as an Antitumor Agent: Disulfiram-Mediated Carbonic Anhydrase 12 and Anion Exchanger 2 Modulation to Inhibit Cancer Cell Migration
Source: Molecules. 2019 Sep 19;24(18):3409. doi: 10.3390/molecules24183409 (PMC6767608; doi:10.3390/molecules24183409)
Supplement: Supplementary file 1 [file molecules-24-03409-s001.pdf]

Supplementary Figure 1. Hwang et al.

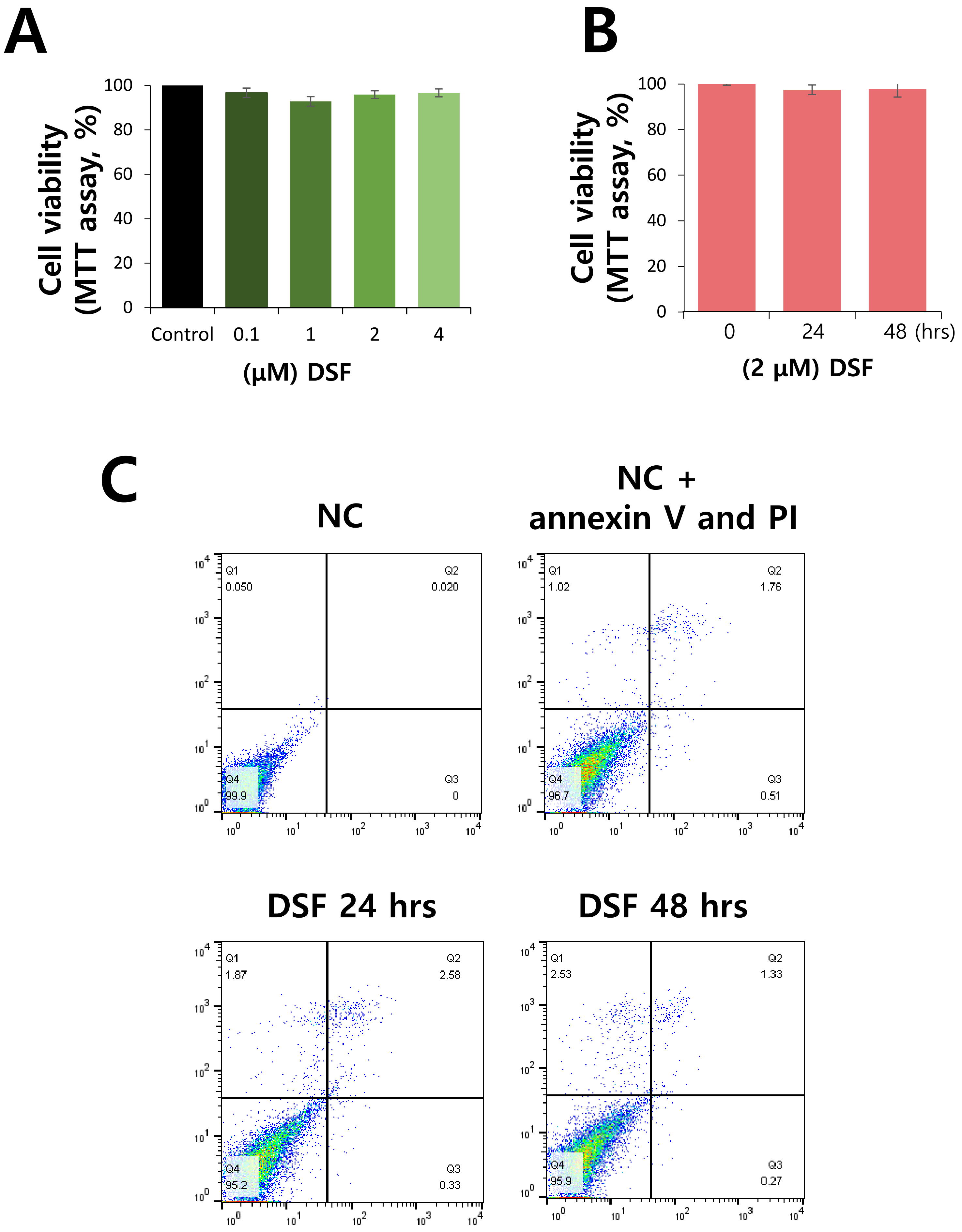

Supplementary Figure 1. Cell viability of DSF-treated A549 cells. Apoptosis analysis using MTT assay**.** The cells were treated with DSF of A549 cells with a dose **(A)** time **(B)**-dependent manner. **(C)** FACS analysis of apoptosis following treatment with 2 μM DSF for indicated time.

Supplementary Figure 2. Hwang et al.

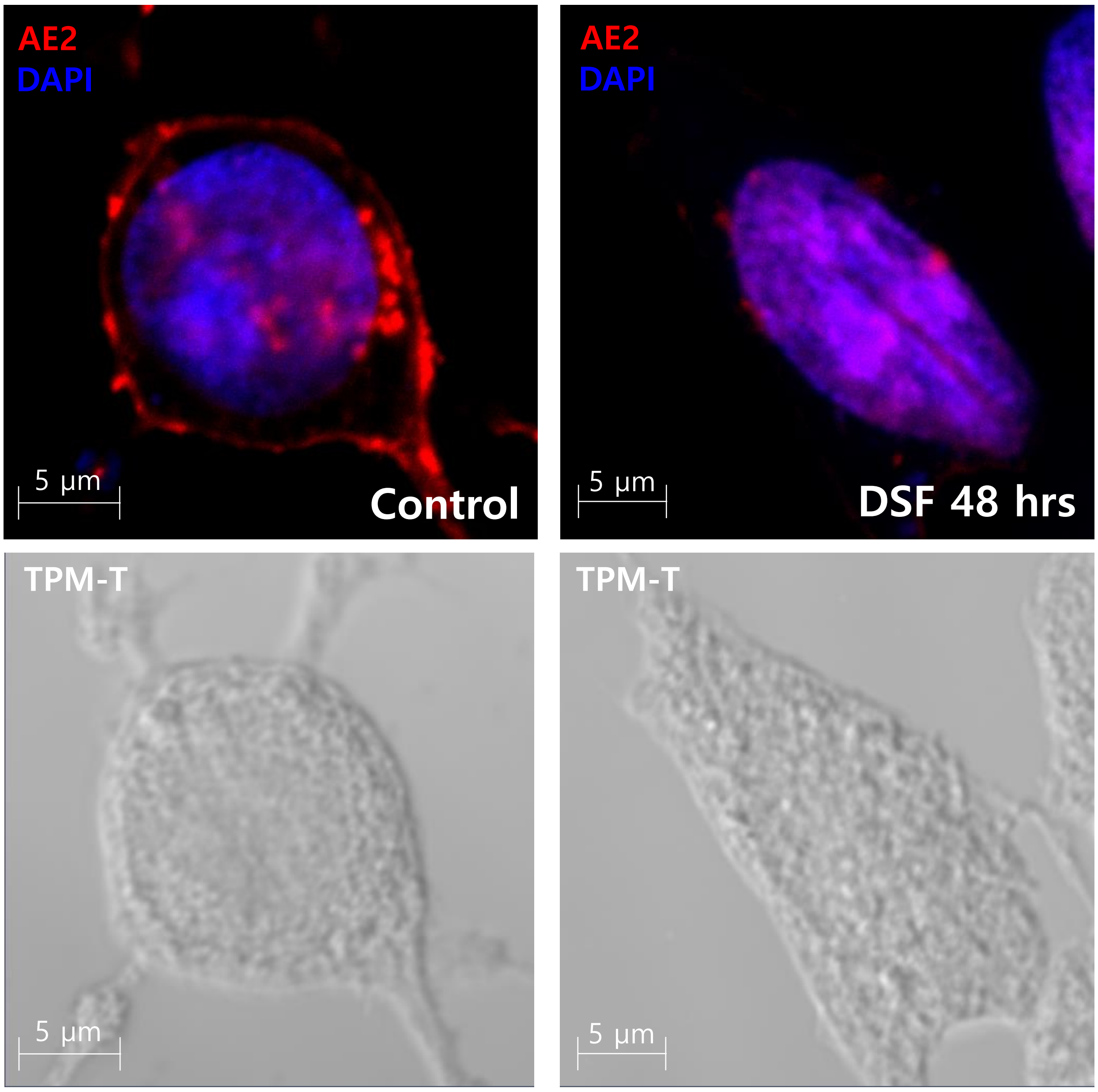

Supplementary Figure 2. The effect of DSF on the localization of AE2 in AE2-overexpressed HEK293T cells. Immunostaining of AE2 (red) and nucleus (DAPI, blue) and cell morphology (TPM-T, gray) following 2 μM DSF treatment for 48 hrs. The scale bars represent 5 μm.

Supplementary Figure 3. Hwang et al.

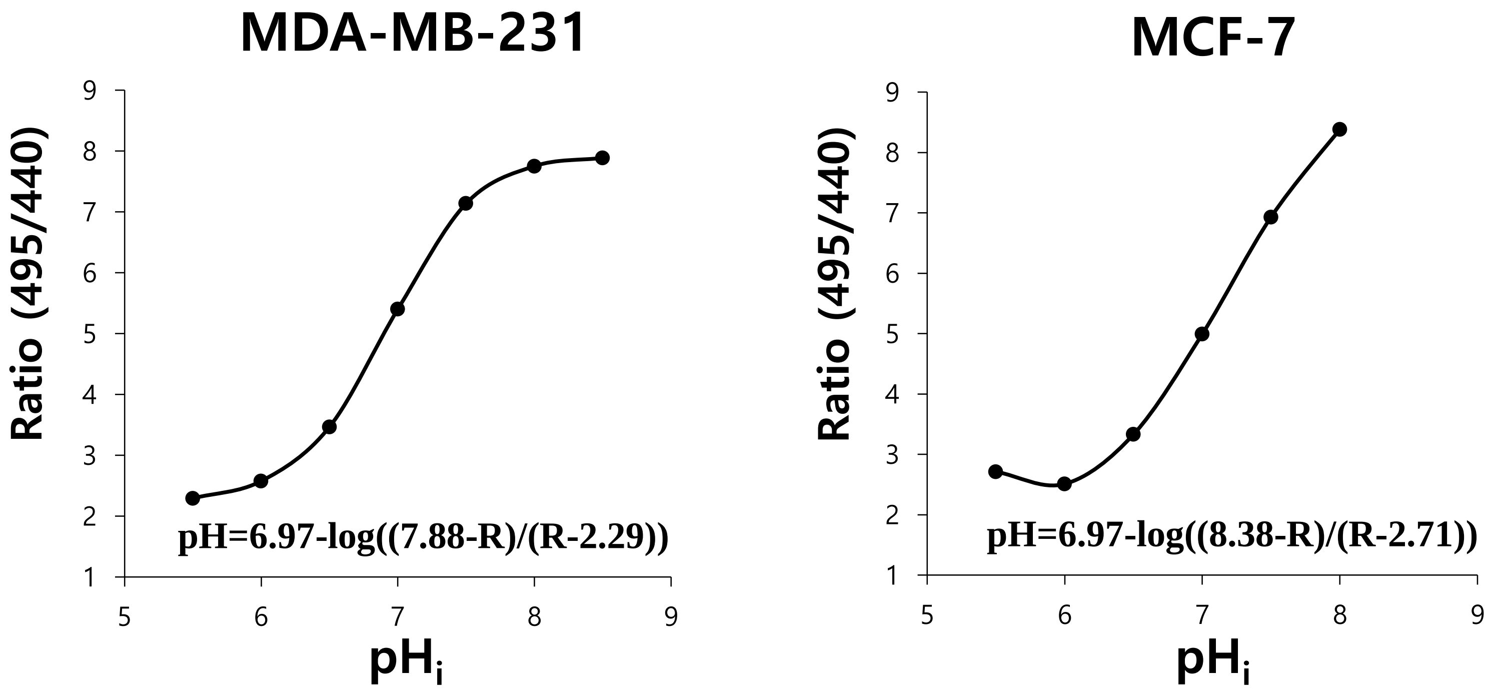

Supplementary Figure 3. Calibration curve of intracellular pH change (ratio) of MDA-MB-231 and MCF-7 cells. Calibration curve showing intracellular pH level of MDA-MB-231 cells (pH = 6.97-log(7.88-R/R-2.29)) and MCF-7 cells (pH = 6.97-log(8.38-R/R-2.71)).
